# Heavy metal biomarkers and their impact on hearing loss risk: a machine learning framework analysis

**DOI:** 10.3389/fpubh.2025.1471490

**Published:** 2025-04-16

**Authors:** Ali Nabavi, Mohammad Kashkooli, Sara Sadat Nabavizadeh, Farimah Safari

**Affiliations:** ^1^Student Research Committee, Shiraz University of Medical Sciences, Shiraz, Iran; ^2^Department of Otolaryngology, Otolaryngology Research Center, Shiraz University of Medical Sciences, Shiraz, Iran

**Keywords:** hearing loss, heavy metals, machine learning, association, risk

## Abstract

**Background:**

Exposure to heavy metals has been implicated in adverse auditory health outcomes, yet the precise relationships between heavy metal biomarkers and hearing status remain underexplored. This study leverages a machine learning framework to investigate these associations, offering a novel approach to understanding the interplay between environmental exposures and hearing loss.

**Methods:**

We conducted a retrospective cross-sectional analysis using data from the 2012–2018 National Health and Nutrition Examination Survey (NHANES), encompassing 2,772 participants after applying exclusion criteria. Demographic, clinical, and heavy metal biomarker data (e.g., blood lead and cadmium levels) were analyzed as features, with hearing loss status—defined as a pure-tone average threshold exceeding 25 dB HL across 500, 1,000, 2000, and 4,000 Hz in the better ear—serving as the binary outcome. Multiple machine learning algorithms, including Random Forest, XGBoost, Gradient Boosting, Logistic Regression, CatBoost, and MLP, were optimized and evaluated. Model performance was assessed using accuracy, area under the curve (AUC), sensitivity, and specificity, while SHAP (SHapley Additive exPlanations) elucidated feature contributions.

**Results:**

The CatBoost model demonstrated the strongest performance, achieving an accuracy of 74.9% and an AUC of 0.792 on test data. Age, education level, gender, and blood levels of lead and cadmium emerged as the most significant features associated with hearing loss, as determined by SHAP analysis. These findings highlight key correlates of hearing impairment within the study population.

**Conclusion:**

This study underscores the utility of a machine learning framework in identifying associations between heavy metal biomarkers and hearing loss in a nationally representative sample. While not designed to forecast hearing loss over time, our findings suggest potential clinical relevance for identifying individuals with elevated heavy metal exposure who may warrant further audiometric evaluation. This work lays a foundation for future longitudinal studies to explore these relationships more comprehensively.

## Introduction

Hearing loss is a critical global concern affecting approximately 27.7 million adults in the United States alone (1, 2). Beyond its prevalence, hearing loss imposes considerable psychological and socioeconomic burdens (3). Evidence accumulated over recent decades suggests the ototoxic effects of heavy metals like lead, cadmium, and mercury, even at low levels of exposure (4, 5). Agricultural, pharmaceutical, industrial settings and certain medical applications are common pathways for heavy metal exposure, with exposure rates rising dramatically in recent decades (6, 7).

Some proposed mechanisms of heavy metal-induced hearing loss include damage to the structures and nervous system of the inner ear, reduced blood flow, and lipid peroxidation in the cochlea (8, 9). A study conducted by Wang et al. proved the relationship between heavy metal exposure and risk of hearing loss through a meta-analysis of recent studies (5). Also, the exacerbating effect of heavy metals even in noise-induced hearing loss has been determined in the Korean population (10). Many animal studies have also demonstrated the correlation and underlying mechanism (11–13). However, no studies to date have utilized machine learning (ML) to formally quantify these relationships and detect hearing loss based on objective exposure biomarker values. Since the screening and diagnostic fields are becoming smarter, an automatic system and platform based on artificial intelligence that investigates hearing loss can be developed to reflect the complex relationship between heavy metals and hearing outcome.

Due to prolonged exposure to heavy metals leading to an increased risk of hearing loss, an accurate investigation tool for high-risk populations enables early intervention and reduces the burden of hearing loss. Artificial intelligence, especially supervised learning which uses labeled inputs and outputs, can develop accurate models to explore the risk of hearing loss. In this study, we try different supervised classification algorithms on a large, nationally representative sample from the National Health and Nutrition Examination Survey (NHANES) dataset to help the model learn the complex relationships between heavy metal levels and other attributes and the risk of hearing loss. As ML models become more complex, it is necessary to have explainable methods that can clarify the contribution of different features to the final result. Feature importance-based explanations have been used to enhance the safety and tractability of the models. We conduct an interpretability analysis to address a key limitation of black box models by explaining the reasons behind their performances. This kind of analysis provides clinicians and researchers more confidence in using the models for risk assessment purposes.

## Method

### Study design and data source

We conducted a retrospective analysis using data from the National Health and Nutrition Examination Survey (NHANES) from 2012 to 2018. NHANES is an ongoing cross-sectional survey conducted by the National Center for Health Statistics to assess the health and nutritional status of adults and children in the United States. The survey combines interviews, physical examinations, and laboratory tests using a complex, multistage probability sampling design to obtain nationally representative samples (14).

**Figure 1:**
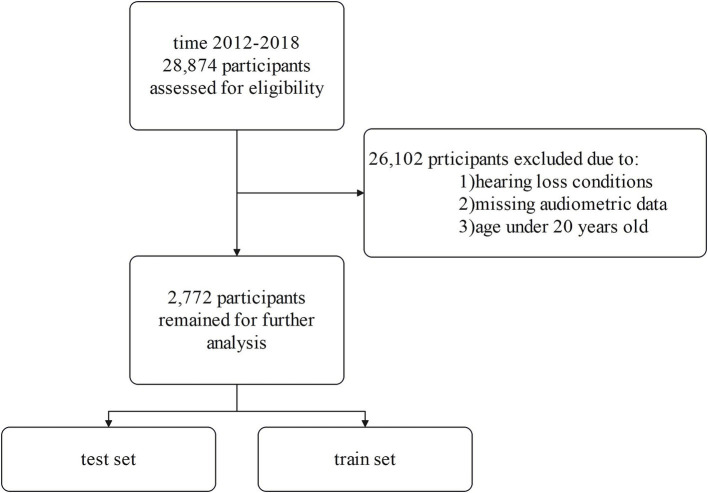
Flow diagram of the cohort study.

From the initial 28,874 NHANES participants, we applied several exclusion criteria to obtain our final analytic sample. We excluded individuals under 20 years of age, those with incomplete audiometric data, and those who self-reported any hearing loss or related conditions. This exclusion criterion was applied to focus the analysis on objectively classified hearing loss based on audiometric data, thereby reducing potential biases from heterogeneous underlying conditions and supporting the study’s aim of identifying associations rather than longitudinal prediction. Participants were excluded if they answered affirmatively to questions about potential causes of hearing loss such as genetic/hereditary factors, ear infections, ear diseases, illnesses/infections, drugs/medications, head/neck injuries, exposure to loud brief noise, long-term noise exposure, or aging. We excluded participants who self-reported significant exposure to loud noise based on the NHANES Audiology Questionnaire. Specifically, individuals who indicated past or current occupational noise exposure or substantial off-work exposure to loud noise were removed from the analysis. To account for the potential impact of ototoxic medications on hearing loss, we reviewed data from the NHANES Prescription Medication Section (DSQ), which records prescription drugs taken by participants in the past 30 days. Based on clinical evidence and prior research, we identified a list of ototoxic medications, including acetaminophen (15), hydrocodone (16), ciprofloxacin (17), phenytoin (18), levofloxacin (19), rifampin (20), minocycline (21), aspirin (22), metronidazole (23), nitroglycerin (24), and bumetanide (25, 26). Only 35 participants (1.3% of the total sample) reported using these medications, and due to the low prevalence of exposure, we retained these individuals in the analysis to avoid unnecessary reduction in sample size and maintain the representativeness of the cohort. After rigorously applying these exclusion criteria, our final analytic sample consisted of 2,772 eligible NHANES participants (Figure 1).

To develop our model for hearing impairment, we selected demographic variables (gender, age, race/ethnicity, education level, marital status, family income-to-poverty ratio), clinical measures (blood pressure, physical activity, smoking status, diabetes diagnosis, body mass index, health insurance status), and biomarkers of heavy metal exposure (mercury, lead, cadmium, arsenic, barium, cobalt, cesium, molybdenum, manganese, antimony, tin, thallium, tungsten, and uranium) consistently measured in the NHANES dataset from 2012 to 2018. These variables were chosen based on their potential associations with hearing health, as identified through an extensive literature review.

### Hearing loss status

**Figure 2:**
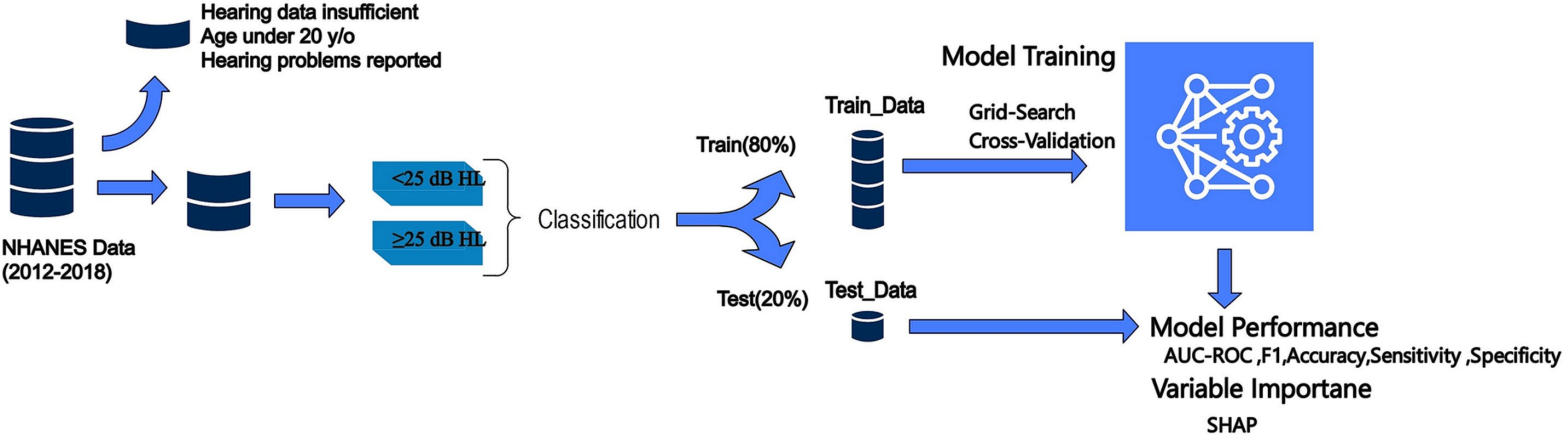
Proposed methodology.

We defined hearing impairment as a binary outcome based on the speech-frequency pure-tone average (PTA), calculated as the mean hearing threshold in decibels hearing level (dB HL) across 500, 1,000, 2000, and 4,000 Hz frequencies for the better ear. This approach aligns with the audiometric testing procedures and guidelines established by the American Speech-Language-Hearing Association. Specifically, we categorized participants as having a hearing impairment (coded as 1) if their PTA value exceeded 25 dB HL, indicating mild or worse impairment. Participants with PTA values below or equal to 25 dB HL were considered to have normal hearing (coded as 0) (27) (Figure 2).

### Preprocessing

Prior to model development, a series of preprocessing steps were undertaken to optimize the NHANES dataset for analysis. Responses indicating refusal or lack of knowledge were considered missing values to mitigate potential biases in performance metrics. A threshold was established to filter out variables and participants with substantial missing information — in particular, variables lacking over 20% of their data and participants missing crucial data points were removed from the study.

To address the presence of outliers, a two-stage detection strategy was employed. Initially, DBSCAN (Density-Based Spatial Clustering of Applications with Noise) was applied to isolate outliers based on density estimations, effectively managing clusters of varying densities. This was complemented by a tree-based anomaly detection method that isolates outliers through random feature partitioning, enhancing robustness in the multidimensional feature space. This dual approach ensured comprehensive outlier management without relying on distributional assumptions, further refining the dataset for analysis.

Categorical and ordinal features were one-hot encoded, transforming them into a machine-readable numerical format, thereby facilitating their inclusion in the modeling process. For numerical variables, scalar normalization techniques were applied to ease the influence of outliers and ensure equitable feature contributions (28).

To address missing data, a hybrid imputation strategy was implemented to enhance dataset robustness. For categorical variables, missing values were imputed using the mode, maintaining their integrity. For numerical variables, an iterative imputation technique was employed, predicting missing values based on relationships with other features across multiple iterations. This approach offers a more sophisticated alternative to mean imputation, minimizing bias and preserving the dataset’s predictive power while ensuring all relevant cases were retained for analysis.

To address missing data, an imputation strategy was implemented, utilizing the mode for categorical variables and the mean for numerical variables. This approach preserved the integrity of the dataset while enabling the inclusion of all relevant cases in the analysis.

Given the dataset’s noticeable imbalance, predominantly skewed toward individuals without hearing impairment, the Synthetic Minority Over-sampling Technique (SMOTE) was implemented. SMOTE enhances model performance on imbalanced data by creating synthetic examples of the under-represented class, thereby balancing the dataset and promoting a more equitable learning environment.

For feature selection, Recursive Feature Elimination (RFE) was utilized in conjunction with a Random Forest estimator. RFE is an iterative process that recursively eliminates the least important features based on the estimator’s feature importance rankings, ultimately retaining the 20 most relevant features for hearing impairment. This approach facilitated the identification of the most significant factors, enhancing the interpretability and performance of the final model.

### Machine learning model evaluation

In our study, we employed a rigorous evaluation process to assess the performance of several machine learning algorithms in exploring hearing outcomes based on heavy-metal exposure. We used Python version 3.8.8. to develop Random Forest, XGBoost, Gradient Boosting, Logistic Regression, CatBoost, and MLP (29–31). These algorithms were selected based on their proven track record in similar tasks across various domains, as demonstrated by their superior performance metrics in related research (32, 33).

To optimize the capabilities of each model, we undertook a comprehensive hyperparameter tuning process. This involved an extensive exploration of the hyperparameter spaces for each algorithm, to identify the configurations that yielded the most accurate performances. We employed a randomized search strategy across 5 iterations, with 5-fold cross-validation for each model. This approach ensured that the models were fine-tuned to their optimal settings for our specific task (Supplementary Table S1).

Our validation methodology involved splitting the dataset into training and test subsets, with 70% of the data allocated for training purposes and the remaining 30% used for model evaluation. The models’ performances were assessed using a comprehensive suite of metrics, including accuracy, sensitivity, specificity, precision, the area under the curve (AUC), and the F1 score. These metrics provided a holistic view of each model’s strength and reliability, taking into account their ability to correctly identify true positives (sensitivity) and true negatives (specificity), as well as their overall accuracy and precision.

### Model interpretation

To enhance the interpretability of these models, we leveraged SHAP (SHapley Additive exPlanations), a powerful framework that provides intuitive explanations by attributing the model’s results to the contributions of individual features (34).

One of the key visualizations we utilized was the SHAP beeswarm plot. These plots aggregate the SHAP values of all features across the dataset, illustrating the distribution of each feature’s impact on the model’s output. The color coding within these plots aids in discerning whether a feature increases or decreases the likelihood of hearing loss, offering a clear understanding of the feature’s influence.

Furthermore, we employed SHAP decision plots to gain a granular understanding of the decision-making process for individual outcomes. These plots trace the path from the base value (the model’s output value without any feature information) to the final performance, sequentially adding the effect of each feature. This step-by-step breakdown provides a transparent narrative of how each feature contributes to the outcome, highlighting the complex interactions and nonlinear relationships between features (35).

Integrating SHAP into our analysis bridged the gap between model accuracy and interpretability, allowing for a deeper comprehension of the underlying patterns and relationships within the data.

## Result

### Characteristics of the study population

Among 2,779 participants who retrained for the analysis, 468 (16.88%) contributors had experienced hearing loss. A comparative analysis between the hearing loss and no hearing loss groups revealed significant differences in their baseline characteristics, as presented in Tables 1, 2. Participants with hearing loss tended to be older, smokers, with lower educational levels and family income, and a higher prevalence of hypertension and diabetes (all *p* < 0.001). The proportion of male participants decreased from 63.03% in the hearing loss group to 47.74% in the no hearing loss group, suggesting potential gender differences between the groups (*p* < 0.001).

**Table 1:** Baseline numerical characteristics of the participants.

Numerical variable	Total, mean (SD)	Hearing loss, mean (SD)	No hearing loss, mean (SD)	*p-*value
Age	45.267 (15.517)	59.464 (12.598)	42.389 (14.433)	<0.001
BMI	29.378 (7.156)	29.897 (6.725)	29.273 (7.237)	0.071
Minutes vigorous-intensity work	52.784 (117.619)	62.006 (128.742)	51.215 (115.587)	0.178
Minutes moderate-intensity work	85.699 (133.125)	89.816 (134.650)	84.999 (132.891)	0.570
Minutes moderate recreational activities	38.126 (56.087)	33.173 (47.906)	38.969 (57.333)	0.063
Minutes severe recreational activities	23.005 (53.645)	30.680 (69.828)	21.700 (50.287)	0.035
Mercury, Inorganic, blood (ug/L)	0.272 (0.333)	0.265 (0.280)	0.272 (0.343)	0.642
Mercury, ethyl, blood (ug/L)	0.111 (0.035)	0.104 (0.032)	0.112415 (0.036)	<0.001
Lead, blood (ug/dL)	1.304 (1.575)	1.663 (1.718)	1.230 (1.534)	<0.001
Cadmium, blood (ug/L)	0.489 (0.568)	0.530 (0.576)	0.481 (0.567)	<0.001
Mercury, total, blood (ug/L)	1.631 (2.882)	1.573 (3.066)	1.643 (2.843)	0.651
selenium, blood (ug/L)	193.766 (25.134)	194.142 (27.118)	193.689 (24.714)	0.741
Manganese, blood (ug/L)	10.124 (3.963)	9.871 (3.933)	10.176 (3.968)	0.131
Arsenous acid, urine (ug/L)	0.442 (0.440)	0.390 (0.399)	0.453 (0.447)	<0.001
Arsenic acid, urine (ug/L)	0.611 (0.180)	0.610 (0.170)	0.611 (0.182)	0.839
Arsenobetaine, urine (ug/L)	10.895 (42.049)	9.950 (39.342)	11.086 (42.583)	0.575
Arsenocholine, urine (ug/L)	0.206 (0.604)	0.190 (0.299)	0.210 (0.649)	0.307
Dimethylarsinic acid, urine (ug/L)	5.582 (7.380)	5.545 (8.868)	5.590 (7.042)	0.918
Monomethylarsonic acid, urine (ug/L)	0.684 (0.589)	0.605 (0.507)	0.700 (0.603)	<0.001
Mercury, urine (ug/L)	0.486 (1.397)	0.514 (2.600)	0.481 (0.991)	0.784
Barium, urine (ug/L)	1.771 (3.001)	1.809 (2.552)	1.763 (3.085)	0.735
Cadmium, urine (ug/L)	0.328 (0.417)	0.418 (0.416)	0.310 (0.414)	<0.001
Cobalt, urine (ug/L)	0.495 (0.686)	0.502 (0.688)	0.494 (0.686)	0.810
Cesium, urine (ug/L)	4.945 (3.914)	4.938 (3.100)	4.946 (4.060)	0.959
Molybdenum, urine (ug/L)	50.659 (46.275)	49.089 (41.264)	50.978 (47.231)	0.379
Manganese, urine (ug/L)	0.164 (0.563)	0.149 (0.200)	0.167 (0.611)	0.240
Lead, urine (ug/L)	0.540 (1.031)	0.632 (1.000)	0.521 (1.036)	<0.001
Antimony, urine (ug/L)	0.079 (0.303)	0.066 (0.082)	0.082 (0.330)	0.030
Tin, urine (ug/L)	1.159 (3.210)	1.428 (3.388)	1.104 (3.170)	0.571
Thallium, urine (ug/L)	0.198 (0.201)	0.177 (0.129)	0.203 (0.213)	<0.001
Tungsten, urine (ug/L)	0.129 (0.664)	0.095 (0.142)	0.136 (0.725)	0.013
Arsenic, urine (ug/L)	18.518 (48.018)	17.219 (44.624)	18.781 (48.682)	0.497
Uranium, urine (ug/L)	0.009 (0.016)	0.009 (0.013)	0.009 (0.017)	0.638

**Table 2:** Baseline categorical characteristics of the participants.

Categorical variable		Total, *N* (%)	Hearing loss, *N* (%)	No hearing loss, *N* (%)	*p* value
Gender	Male	1,395 (50.32)	295 (63.03)	1,100 (47.74)	<0.001
	Female	1,377 (49.68)	173 (36.97)	1,204 (52.26)	
Race/Ethnicity	Mexican American	378 (13.64)	68 (14.53)	310 (13.46)	0.009
	Other Hispanic	340 (12.27)	64 (13.68)	276 (11.98)	
	Non-Hispanic White	918 (33.11)	179 (38.25)	739 (32.07)	
	Non-Hispanic Black	663 (23.92)	89 (19.02)	574 (24.91)	
	Other Race	473 (17.06)	68 (14.52)	405 (17.58)	
Education level	Less than 9th grade	234 (8.44)	71 (15.20)	163 (7.07)	<0.001
	9–11th grade	318 (11.48)	82 (17.56)	236 (10.24)	
	High school graduate/GED	604 (21.80)	121 (25.91)	483 (20.96)	
	Some college or AA degree	848 (30.60)	110 (23.55)	738 (32.03)	
	College graduate or above	767 (27.68)	83 (17.77)	684 (29.69)	
Family PIR	<1.30	831 (29.98)	155 (33.12)	645 (27.99)	<0.001
	1.30–3.49	943 (34.02)	164 (35.04)	760 (32.99)	
	>3.50	998 (36.00)	149 (31.84)	899 (39.02)	
Marital status	Married	1,394 (50.32)	266 (56.96)	1,128 (48.98)	<0.001
	Widowed	112 (4.04)	46 (9.85)	66 (2.87)	
	Divorced	276 (9.96)	67 (14.35)	209 (9.08)	
	Separated	87 (3.15)	14 (3.00)	73 (3.17)	
	Never married	611 (22.06)	47 (10.06)	564 (24.49)	
	Living with partner	290 (10.47)	27 (5.78)	263 (11.41)	
Health insurance	Yes	2,175 (78.55)	398 (85.04)	1777 (77.23)	<0.001
	No	594 (21.45)	70 (14.96)	524 (22.77)	
Smoking	Never smoker	1,580 (57.00)	252 (53.85)	1,336 (57.99)	<0.001
	Past smoker	554 (19.99)	102 (21.80)	437 (18.97)	
	Current smoker	638 (23.02)	114 (24.35)	531 (23.04)	
Hypertension	Yes	872 (31.47)	227 (48.5)	645 (28.01)	<0.001
	No	1899 (68.53)	241 (51.5)	1,658 (71.99)	
Diabetes	Yes	344 (13.20)	121 (27.01)	223 (9.86)	<0.001
	No	2,362 (86.80)	327 (72.99)	2035 (90.14)	

Blood sample analysis showed significantly higher levels of lead, cadmium, and ethylmercury in the hearing loss group (all *p* < 0.001). Furthermore, urine samples from the same group exhibited considerably higher levels of lead, cadmium, arsenous acid, thallium, antimony, tungsten, and monomethylarsonic acid (all *p* < 0.001).

### Machine learning model performance

**Figure 3:**
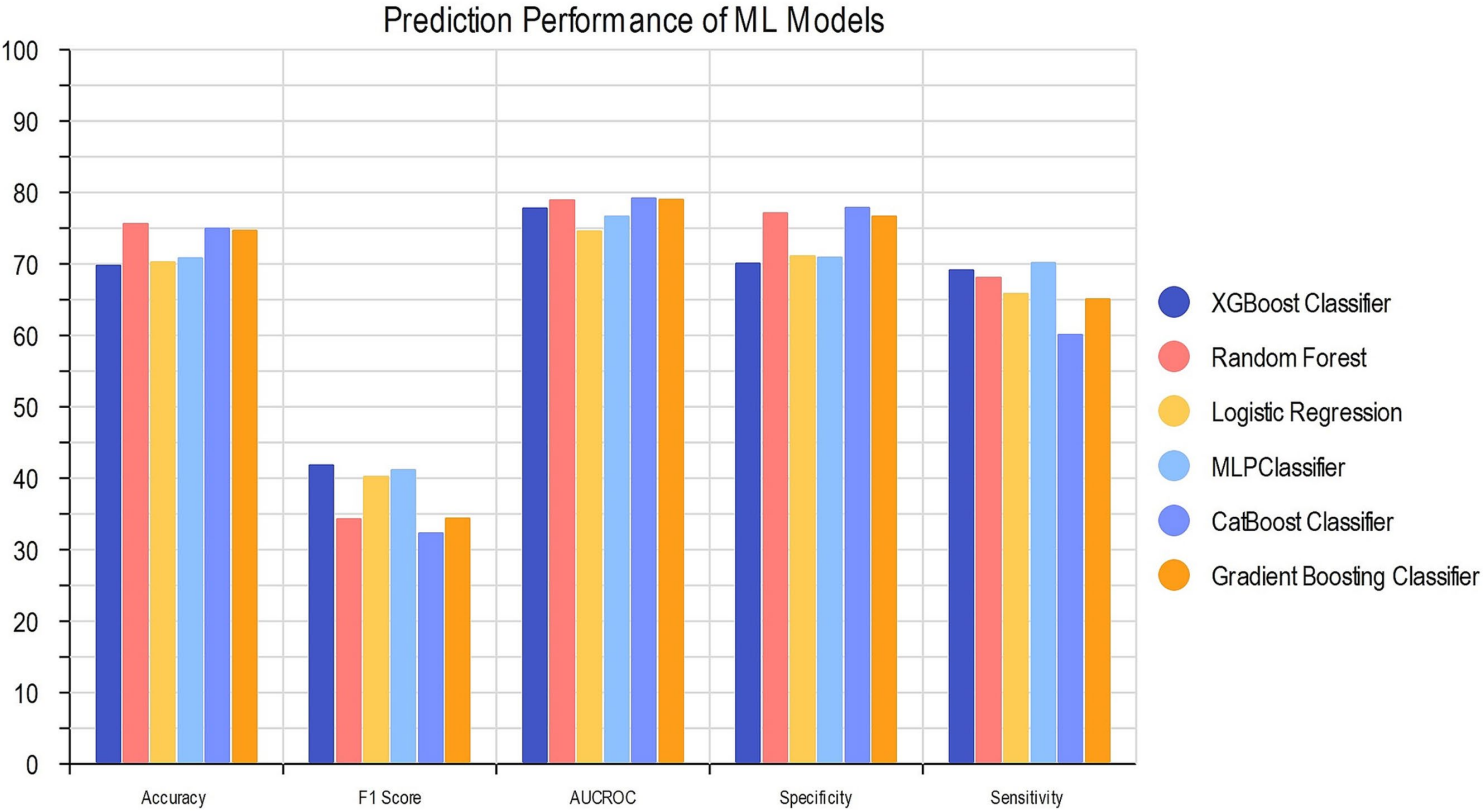
Comparison of ML models’ performance.

We compared the performance of our six ML algorithms which were each trained and tested precisely. To prevent overfitting or uncertainty in the models, we utilized RFE to penalize and select our 22 optimal features for model development. The details of models’ performance metrics are presented in Table 3 and the visualized comparison between ML models is shown in Figure 3.

**Table 3:** Prediction performance of ML models.

Model		Accuracy	F1 score	AUCROC	Specificity	Sensitivity
RF	Train set	0.756 (0.754–0.757)	0.343 (0.339–0.348)	0.789 (0.779–0.800)	0.771 (0.768–0.773)	0.681 (0.678–0.683)
	Test set	0.756	0.348	0.772	0.768	0.696
XGBoost	Train set	0.698 (0.695–0.700)	0.418 (0.415–0.424)	0.778 (0.774–0.780)	0.700 (0.698–0.701)	0.691 (0.690–0.692)
	Test set	0.747	0.309	0.750	0.786	0.577
CatBoost	Train set	0.749 (0.739–0.761)	0.323 (0.320–0.325)	0.792 (0.790–0.793)	0.779 (0.778–0.780)	0.601 (0.600–0.601)
	Test set	0.740	0.342	0.786	0.764	0.621
Gradient Boosting	Train set	0.747 (0.745–0.750)	0.344 (0.340–0.350)	0.790 (0.789–0.792)	0.766 (0.765–0.768)	0.651 (0.650–0.653)
	Test set	0.734	0.347	0.762	0.758	0.619
Logistic Regression	Train set	0.702 (0.701–0.704)	0.402 (0.400–0.403)	0.746 (0.744–0.749)	0.711 (0.707–0.716)	0.658 (0.656–0.659)
	Test set	0.681	0.401	0.723	0.698	0.597
MLP	Train set	0.708 (0.705–0.710)	0.411 (0.409–0.412)	0.766 (0.765–0.768)	0.709 (0.701–0.715)	0.701 (0.699–0.702)
	Test set	0.708	0.385	0.749	0.723	0.632

**Figure 4:**
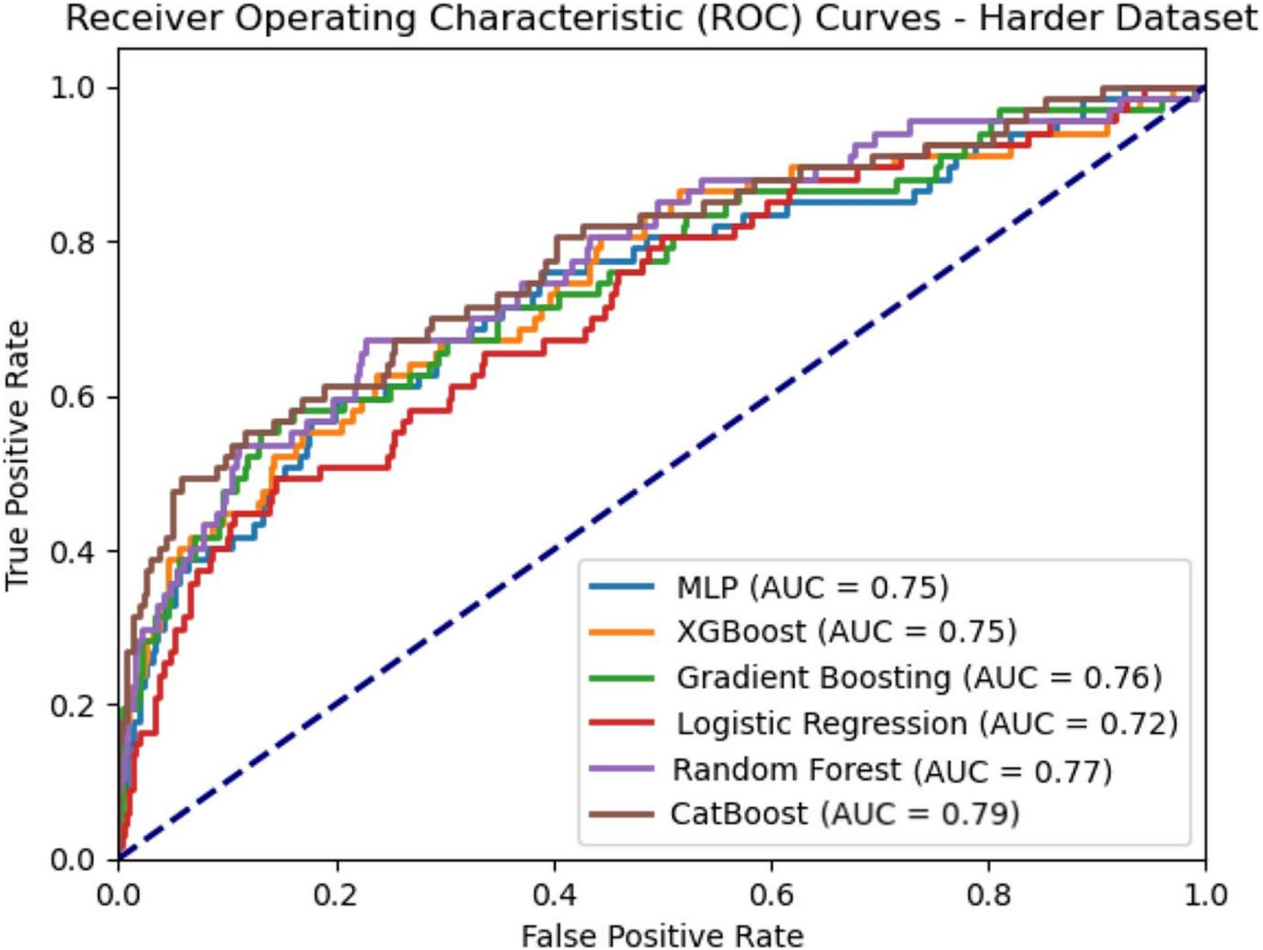
AUC-ROC curves of ML models.

CatBoost classifier outperformed other models with an accuracy of 74.9 and 74.0% and AUC of 0.792 and 0.786 for train and test groups. Compared to Logistic Regression, our best model achieved a satisfactory increase of more than 4% in AUC and accuracy. Among all six models, XGBoost had the best precision with 0.786 for test group, while the best sensitivity belongs to MLP with 0.701 for train group. The results of the AUC metric are depicted in Figure 4, which shows the averaged ROC curves across the full range of specificity and sensitivity thresholds for all six models.

### Feature importance

**Figure 5:**
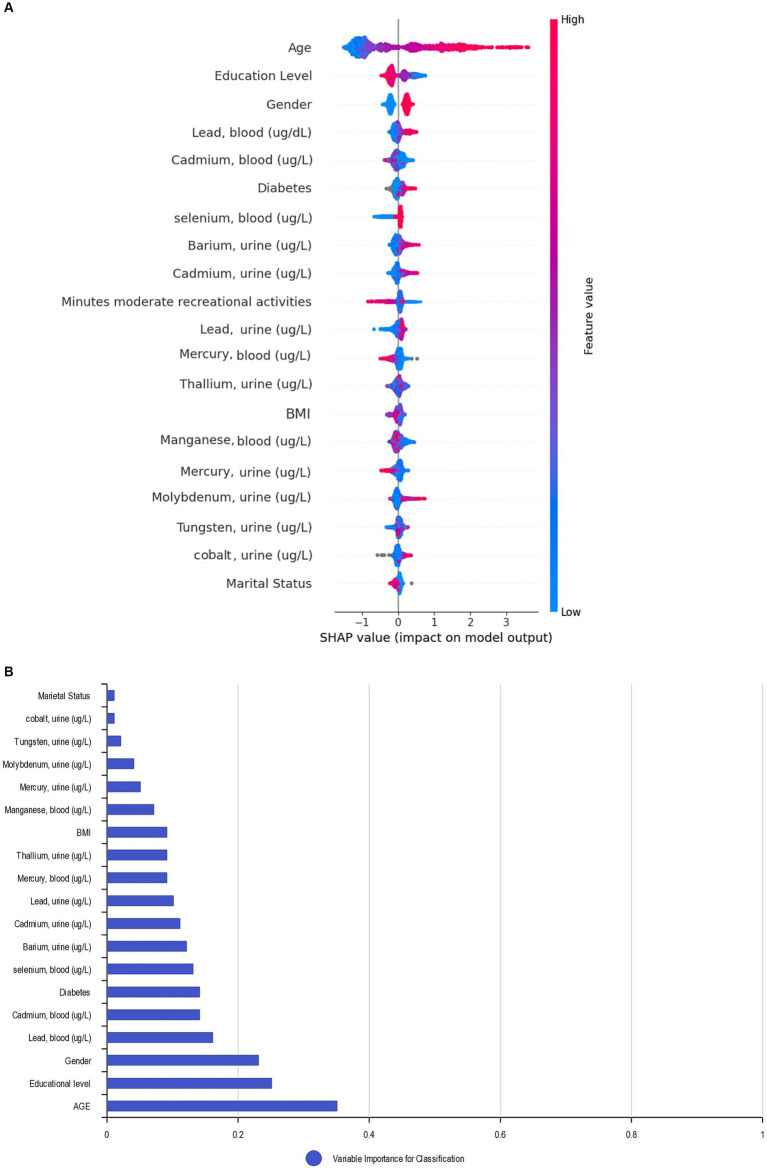
SHAP BeeSwarm plot and feature importance. **(A)** SHAP BeeSwarm plot for detecting hearing loss in the CatBoost model. **(B)** Feature importance ranking for detecting hearing loss in the CatBoost model. ug/L: Mean concentration; BMI: Body mass index.

We applied the Bee Swarm SHAP method to explain the role of each feature and the effect on the model for detecting hearing loss in the CatBoost model, where the red and blue features represent associated factors and protective factors, respectively (Figure 5A). Also, in terms of SHAP values, longer bars meant more importance. Additionally, we sorted the importance of variables in ascending order according to the average value as presented in Figure 5B. The result shows that age was the most influential associated factor for HL classification, followed by lower educational levels. Male gender was also positively correlated with hearing loss which made it more likely that the model classifies the participant as a hearing loss case. Among heavy metals, higher blood lead and cadmium levels were the most important associated factors, while selenium and barium were other important heavy metals with lower impact on positive association.

**Figure 6:**
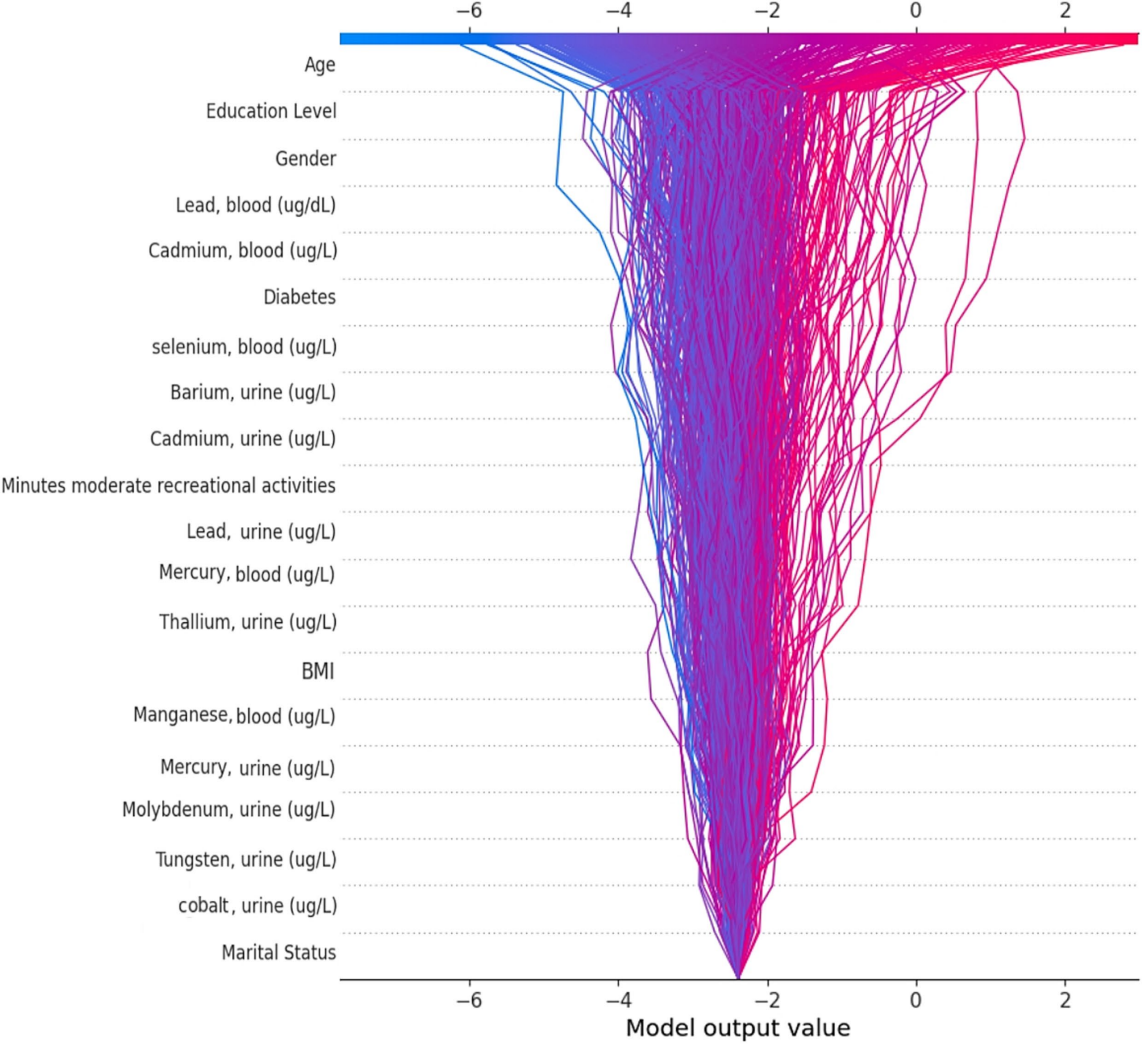
SHAP decision plot.

By utilizing the SHAP decision plot, we tried to clarify how the CatBoost model arrived at the outcome for each particular instance. As shown in Figure 6, each line represents a participant in the decision plot. Lines toward the right side mean that features like older age, higher blood lead levels, and less education pushed the model result toward hearing loss. Conversely, lines toward the left indicate that features like female gender, nonsmoking status, and lower selenium levels led the model to demonstrate no hearing loss.

## Discussion

We evaluated six ML models to expedite early detection of hearing loss caused by heavy metal exposure. Our study used a representative NHANES dataset sample to identify the model with the highest classification accuracy. An estimated AUC in the range of 0.7–0.8 in our models is generally considered a “Good” capacity (36). The CatBoost algorithm, the best-performing model, attained a notable accuracy of 74.9% and an AUC of 0.792. Besides, the SHAP algorithm identified age, education level, gender, lead, and cadmium concentration as the main features that contributed to the conclusion. The timely identification of hearing loss through the application of this screening tool facilitates early intervention and treatment, thereby enhancing the quality of life.

Recently many studies have utilized ML to examine hearing outcomes based on various features. An AUC of 0.93 and accuracy of 85% based on speech-in-noise testing, an AUC of 0.80 and accuracy of 80% based on noise exposure, and an AUC of 0.96 based on demographics and clinical outcomes are some of the best examples of exploring hearing outcome. However, we believe our study represents a pioneering effort to utilize heavy metals as associated factors of hearing outcomes (37–39). SVM, RF, and LightGBM were the best models in recent studies, while, almost none of them used the CatBoost algorithm in their study. In addition, recent studies mainly focused on minimizing the absolute error rate and maximizing the precision. While having a precise performance and minimizing error across all cases is statistically favorable, it does not necessarily translate to the best real-world results in a medical setting. If a model is overly focused on precision and mean error, it risks being too conservative in its performance. As a result, patients who do need additional follow-up may be missed. This could have serious consequences for individuals’ long-term hearing health and quality of life. Therefore, instead of focusing solely on precision, we have opted to optimize sensitivity and recall along with accuracy and AUC. This approach is more appropriate for diagnostic evaluations for hearing, which are relatively simple, inexpensive, and low-risk. By combining ML screening with standardized diagnostic testing only for flagged individuals, populations can be efficiently monitored for hearing loss.

The CatBoost algorithm provided our first aim to find the best ML model performance. This particular classifier is known for its ability to effectively manage categorical variables through the application of Ordered Boosting, a variant of gradient boosting. The algorithm’s use of multiple weak models (decision trees) to create a strong ensemble model results in a high level of accuracy, which is particularly useful for capturing nonlinear patterns in data (40). While our primary objective was to develop the most effective hearing loss classification algorithm based on heavy metal exposure, we were also interested in understanding the key factors that influence the decision-making process of these classifiers. To this end, we opted to utilize the SHAP algorithm, as it allows for a greater degree of transparency and interpretability in our model outputs.

The SHAP analysis assigns a value to each feature, which denotes its contribution to the outcome. By using this technique, we can obtain insights into the contribution of each feature in noticing hearing loss for a particular patient (local interpretation), the accuracy of the result for that patient (local explanation), and the contribution of each feature to the model’s performance across the entire dataset (global explanation and interpretation) (41). To provide better global and local insights, and to avoid focusing solely on the global interpretation of Bee Swarm SHAP, we utilized the SHAP decision plot. This plot helps to show the impact of each feature on the model’s output for a specific instance, thus providing a clearer understanding of the diagnosis and reasons behind it.

Our models retained known associated factors from patient characteristics such as increasing age, worse education level, and male gender. Aging is the primary associated factor for hearing loss, while lower education levels are associated with poorer healthcare and greater exposure to the related factors (42, 43). We have also identified being male as a significant associated factor for hearing loss, likely due to increased exposure to occupational and environmental noise that men face on average, as well as biological differences resulting from male sex hormones and their potential effects on hearing vulnerability compared to female hormones (44, 45). In addition, blood lead and cadmium concentrations have been shown to impact hearing outcomes due to their toxic effects on the cochlea and auditory nerve (46, 47). However, urine lead and cadmium appear to have a lower impact on hearing outcomes due to their nature as daily exposure indicators.

Heavy metals, including lead, cadmium, mercury, barium, arsenic, and manganese, have been conclusively linked to hearing loss. Exposure to these heavy metals through various routes, such as diet, inhalation, or dermal absorption, can increase hearing loss by anywhere from 3 to 75% (5, 48). Heavy metals can damage both the axons and myelin of peripheral nerves, with small nerve fibers being more susceptible than their larger counterparts (49). Furthermore, heavy metals can induce oxidative stress, inflammation, and vascular damage in the inner ear (48). The combination of these mechanisms can explain the association between heavy metal exposure and hearing loss. Yang et al. (50) demonstrated a significant association between blood lead levels and hearing loss in their meta-analysis, reinforcing the evidence that heavy metal exposure plays a role in auditory health. Our study builds on this foundation by employing machine learning and SHAP-based analysis to classify hearing status, thereby offering a more detailed, data-driven interpretation of how heavy metals, particularly lead and cadmium, influence hearing impairment.

The ML models developed and evaluated in this study show promise as effective screening tools to help address the growing problem of heavy metal-induced hearing loss. This screening approach could lighten the burden on healthcare systems when prospectively implemented, enabling the efficient monitoring of large populations. By facilitating early detection, the models may help improve long-term patient outcomes by allowing for timely clinical intervention that is tailored to an individual’s risk profile. The non-invasive nature of ML screening makes it a suitable first-line assessment prior to more resource-intensive diagnostic evaluations.

This study is the first of its kind to develop ML models incorporating heavy metal biomarker data in order to discover hearing loss risk. By analyzing which features like lead and cadmium levels contributed most to model outcomes, our study provides a novel perspective on their relative importance in auditory function. This pioneering work lays the foundation for more comprehensively understanding the relationship between environmental exposures and hearing outcomes through the use of advanced analytical techniques. The examination of a high-quality population-based registry was conducted to ensure the accuracy of the outcome. It appears more reasonable to consider this model when selecting high-risk patients rather than ruling out low-risk ones, considering the high recall and low precision. This approach can help capture all high-risk participants due to the availability of diagnosis approaches. However, the study has some limitations that require careful consideration. First, due to the cross-sectional nature of the dataset and lack of follow-up, the causality between the features and hearing loss could not be determined. Second, the use of a single retrospective dataset may lead to confounding effects of unmeasured factors, thereby making it difficult to apply this model to other institutions. Nonetheless, this study provides a foundation for developing a model that fits well with each institution. Third, the analysis of available data was limited by the fact that a significant proportion of participants did not undergo urine testing for cadmium and arsenic. Therefore, further studies are warranted to prospectively examine the detection of hearing loss through heavy metals using a large sample of the general population. Another limitation is the potential confounding effect of ototoxic medications; however, the low prevalence of exposure (*n* = 35, 1.3%) in our cohort suggests minimal impact on results, though future studies with larger samples and detailed medication data, including dosage and long-term use, could provide further insights. Additionally, while exposure to volatile organic compounds (VOCs) and pesticides is known to contribute to hearing loss, their frequent co-occurrence with heavy metals in environmental and occupational settings made it impractical to exclude such participants, as this would have reduced the representativeness of our cohort and introduced selection bias. Due to data limitations, our study could not explicitly account for vibration exposure, a well-established risk factor for hearing loss, which is frequently encountered alongside noise. Future research should also explore the combined effects of noise and vibration on hearing outcomes. While this study made important advances by focusing specifically on investigating hearing loss with heavy metal exposures, future work could aim to achieve even higher performance by integrating these biomarker features within models alongside additional clinical and demographic factors examined in other related research.

## Conclusion

Overall, our findings provide preliminary validation of ML as a decision support tool for monitoring and detecting heavy metal-induced hearing loss at a population level. Specifically, we demonstrate that the CatBoost classifier achieved the highest performance with an AUC of 0.792 and accuracy of 74.9%. With prospective validation in larger, independent cohorts, this framework could aid public health efforts by facilitating early detection efforts and guiding clinical resource allocation.

## Data Availability

Publicly available datasets were analyzed in this study. This data can be found at: National Health and Nutrition Examination Survey (NHANES) repository, https://www.cdc.gov/nchs/nhanes/index.htm.
