# Supplementary material for: Heavy metal biomarkers and their impact on hearing loss risk: a machine learning framework analysis
Source: Front Public Health. 2025 Apr 16;13:1471490. doi: 10.3389/fpubh.2025.1471490 (PMC12040934; doi:10.3389/fpubh.2025.1471490)
Supplement: Supplementary file 1 [file Table_1.docx]

| **Algorithm** | **Hyper-parameter** | **Searching Space** | **Best Parameters**  **For Classification** |
| --- | --- | --- | --- |
| **Linear Regression** | **N/A** | **N/A** |  |
| **Random Forest** | **criterion** | **[gini, entropy]** | **Entropy** |
|  | **n_estimators** | **[10, 50, 100, 200, 500]** | **200** |
|  | **max_depth** | **[5, 10, 15, None]** | **10** |
|  | **max_features** | **['auto', 'sqrt', 'log2']** | **Auto** |
| **Gradient Boosting** | **learning_rate** | **[0.01, 0.1, 0.2, 0.5]** | **0.1** |
|  | **n_estimators** | **[100, 200, 300]** | **100** |
|  | **max_depth** | **[3, 5, 7, 9]** | **5** |
|  | **loss** | **[deviance, exponential]** | **deviance** |
| **XGBoost** | **learning_rate** | **[0.01, 0.1, 0.2, 0.3]** | **0.1** |
|  | **n_estimators** | **[100, 200, 500, 1000]** | **200** |
|  | **max_depth** | **[3, 6, 9, 12]** | **9** |
| **CatBoost** | **learning_rate** | **[0.01, 0.05, 0.1, 0.2]** | **0.05** |
|  | **n_estimators** | **[100, 200, 500, 1000]** | **100** |
|  | **depth** | **[4, 6, 8, 10]** | **4** |
|  | **loss_function** | **[Logloss, CrossEntropy]** | **Logloss** |
| **MLP** | **hidden_layer_sizes** | **[(50,), (100,), (50,50), (100,100)]** | **(50,50)** |
|  | **activation** | **[relu, tanh, logistic]** | **relu** |
|  | **learning_rate_init** | **[0.001, 0.01, 0.1]** | **0.1** |

**Table S1. Range of the evaluated hyperparameters for the machine learning models**
